# Supplementary material for: Killer prey: Ecology reverses bacterial predation
Source: PLoS Biol. 2024 Jan 23;22(1):e3002454. doi: 10.1371/journal.pbio.3002454 (PMC10805292; doi:10.1371/journal.pbio.3002454)
Supplement: S1 Fig — Percentage reduction of P. fluorescens population size after 4 days in the presence of M. xanthus relative to in the absence of M. xanthus. Colors correspond to 3 independent replicates each run with 2 technical replicates. The dataset for this figure and the R script used to analyze it and make the figure are available on Zenodo (10.5281/zenodo.10214013). (PDF) [file pbio.3002454.s001.pdf]

S1 Fig

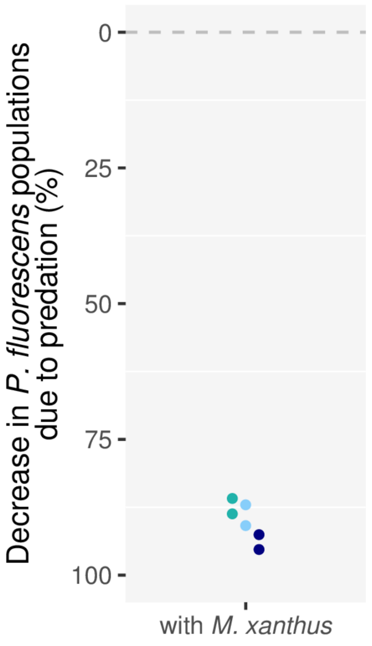

***M. xanthus* kills *P. fluorescens* grown on M9cas agar at 32 °C.** Percentage reduction of *P. fluorescens* population size after four days in the presence of *M. xanthus* relative to in the absence of *M. xanthus*. Colors correspond to three independent replicates each run with two technical replicates. The dataset for this figure and the R script used to analyze it and make the figure are available on Zenodo (10.5281/zenodo.10214013).
