# Supplementary material for: Killer prey: Ecology reverses bacterial predation
Source: PLoS Biol. 2024 Jan 23;22(1):e3002454. doi: 10.1371/journal.pbio.3002454 (PMC10805292; doi:10.1371/journal.pbio.3002454)
Supplement: S2 Fig — M. xanthus population sizes 30 minutes after inoculation onto P. fluorescens lawns grown overnight from one of 3 inoculum sizes and at one of 3 temperatures (green dots) or onto bacteria-free control plates that had been incubated overnight at one of the same 3 temperatures prey (black dots). Means of log10-transformed CFU + 1 values and 95% confidence intervals are shown. Note that, due to a technical issue with dilution plating for 1 replicate of the 12°C treatment, the highest available plated dilution was too low to accurately count colonies; the corresponding plates had more colonies than could feasibly be counted. We therefore attributed counts of 1,000 for these plates, which was clearly a substantial underestimate in each case. These underestimated values are identified in the graph with red circles around corresponding data points. Lighter dots are biological replicates (n = 3). The dataset for this figure and the R script used to analyze it and make the figure are available on Zenodo (10.5281/zenodo.10214013). (PDF) [file pbio.3002454.s002.pdf]

S2 Fig

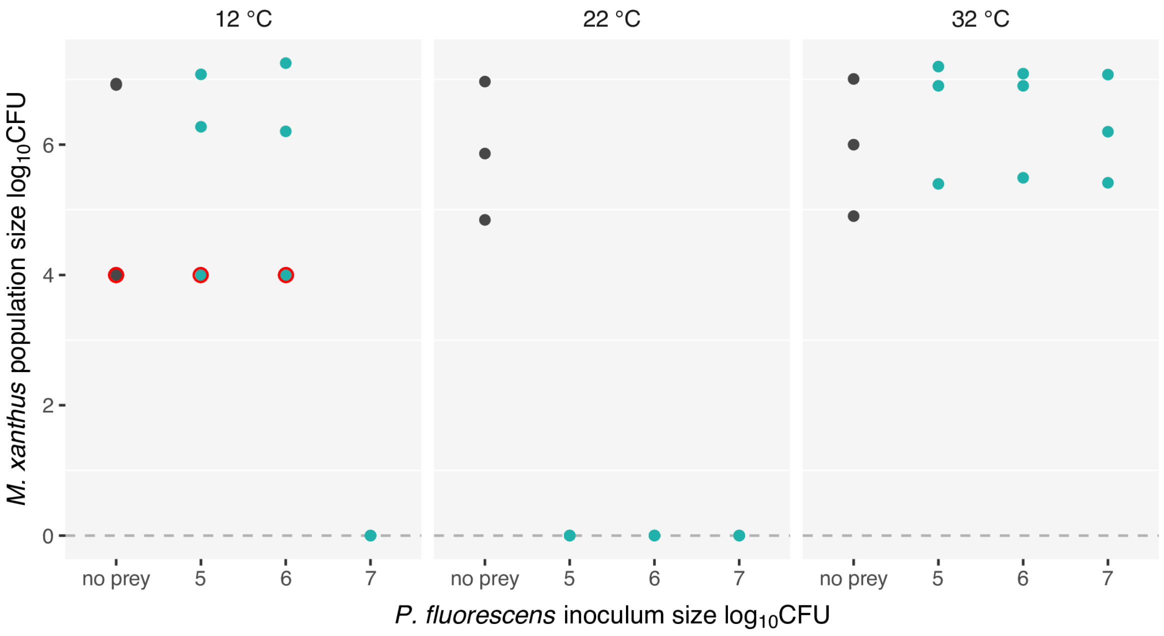

***P. fluorescens* grown at 22 °C kills *M. xanthus* after 30 minutes of interaction.** *M. xanthus* population sizes 30 minutes after inoculation onto *P. fluorescens* lawns grown overnight from one of three inoculum sizes and at one of three temperatures (green dots) or onto bacteria-free control plates that had been incubated overnight at one of the same three temperatures prey (black dots). Means of log_{10}-transformed CFU + 1 values and 95% confidence intervals are shown. Note that, due to a technical issue with dilution-plating for one replicate of the 12 °C treatment, the highest available plated dilution was too low to accurately count colonies; the corresponding plates had more colonies than could feasibly be counted. We therefore attributed counts of 1000 for these plates, which was clearly a substantial underestimate in each case. These underestimated values are identified in the graph with red circles around corresponding data points. Lighter dots are biological replicates (*n* = 3). The dataset for this figure and the R script used to analyze it and make the figure are available on Zenodo (10.5281/zenodo.10214013).
