# Supplementary material for: Killer prey: Ecology reverses bacterial predation
Source: PLoS Biol. 2024 Jan 23;22(1):e3002454. doi: 10.1371/journal.pbio.3002454 (PMC10805292; doi:10.1371/journal.pbio.3002454)
Supplement: S3 Fig — Relationship between P. fluorescens initial and final population sizes after 22 hours of growth at different temperatures 12, 22, or 32°C. Log10-transformed CFU values (n = 3), linear fits, and 95% confidence intervals about the linear fits are shown. The dataset for this figure and the R script used to analyze it and make the figure are available on Zenodo (10.5281/zenodo.10214013). (PDF) [file pbio.3002454.s003.pdf]

S3 Fig

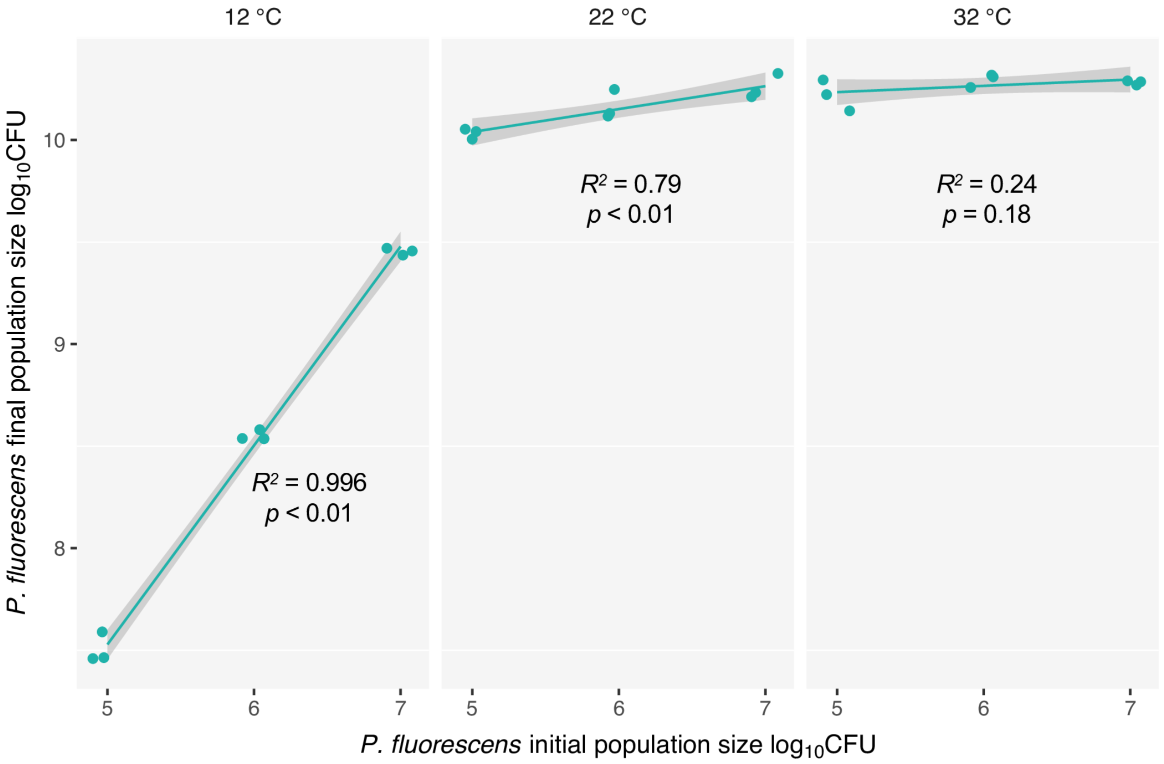

Final *P. fluorescens* population size correlates positively with initial population size after growth at 12 and 22 °C but not at 32 °C. Relationship between *P. fluorescens* initial and final population sizes after 22 hours of growth at different temperatures 12, 22 or 32 °C. Log_{10}-transformed CFU values (n = 3), linear fits and 95% confidence intervals about the linear fits are shown. The dataset for this figure and the R script used to analyze it and make the figure are available on Zenodo (10.5281/zenodo.10214013).
