# Supplementary material for: Killer prey: Ecology reverses bacterial predation
Source: PLoS Biol. 2024 Jan 23;22(1):e3002454. doi: 10.1371/journal.pbio.3002454 (PMC10805292; doi:10.1371/journal.pbio.3002454)
Supplement: S4 Fig — DK3470 population sizes are shown 24 hours after interaction with P. fluorescens when the 2 species interact at the same temperature at which both had been reared prior to interaction. Means of log10-transformed CFU + 1 values and 95% confidence intervals are shown. Lighter dots are biological replicates (n = 3). *** p < 0.001 (Tukey-adjusted contrasts) for the difference between M. xanthus population size after interaction with P. fluorescens (green dots) vs. in the control treatment (black dots) when the 2 species were reared and interacted at 22°C. The dataset for this figure and the R script used to analyze it and make the figure are available on Zenodo (10.5281/zenodo.10214013). (PDF) [file pbio.3002454.s004.pdf]

**S4 Fig**

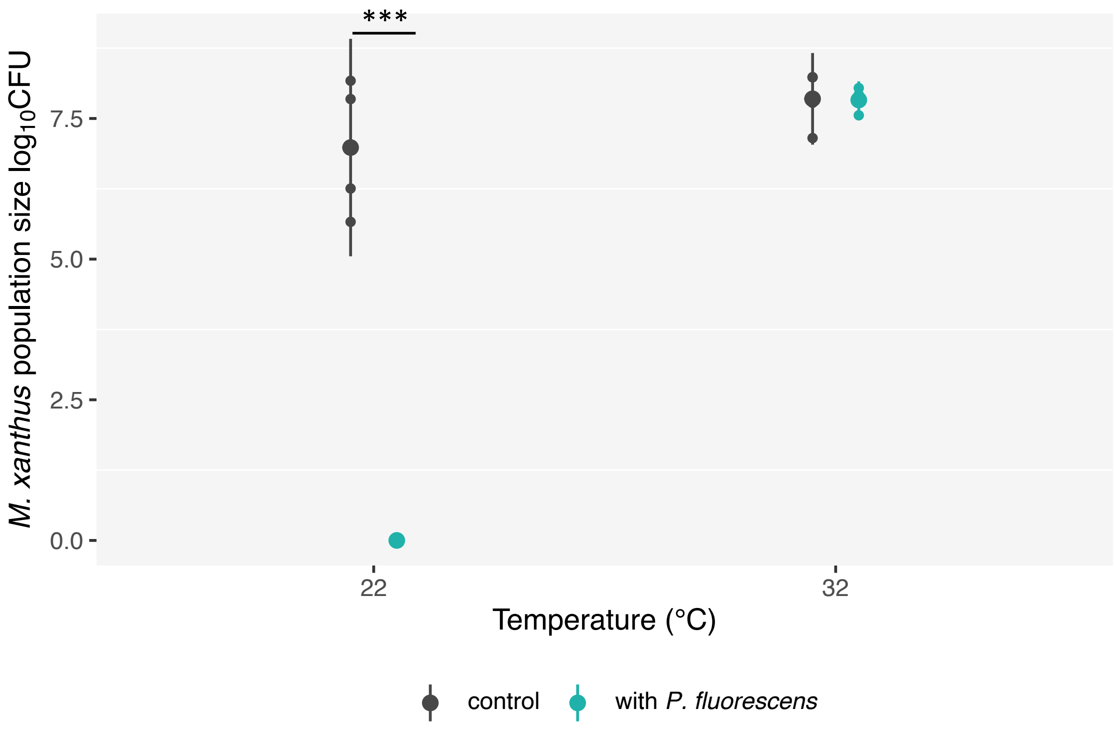


***P. fluorescens* kills *M. xanthus* when both species are pre-grown and interact at 22 °C but not when both are pre-grown and interact at 32 °C.** DK3470 population sizes are shown 24 hours after interaction with *P. fluorescens* when the two species interact at the same temperature at which both had been reared prior to interaction. Means of $log_{10}$-transformed CFU + 1 values and 95% confidence intervals are shown. Lighter dots are biological replicates (n = 3). *** $p < 0.001$ (Tukey-adjusted contrasts) for the difference between *M. xanthus* population size after interaction with *P. fluorescens* (green dots) *vs* in the control treatment (black dots) when the two species were reared and interacted at 22 °C. The dataset for this figure and the R script used to analyze it and make the figure are available on Zenodo (10.5281/zenodo.10214013).
