# Supplementary material for: Killer prey: Ecology reverses bacterial predation
Source: PLoS Biol. 2024 Jan 23;22(1):e3002454. doi: 10.1371/journal.pbio.3002454 (PMC10805292; doi:10.1371/journal.pbio.3002454)
Supplement: S5 Fig — Remains of M. xanthus killed by 22°C-reared P. fluorescens sufficient to fuel large P. fluorescens population growth pass through 0.2-μm filters within 6 hours of interspecies interaction. Estimated densities (log-transformed CFU/ml, n = 3) of P. fluorescens populations over time inoculated at 2 initial densities (approximately 106 and approximately 108 CFU/ml) into supernatant from 22°C-reared P. fluorescens lawns to which M. xanthus cells were either added (and which killed those M. xanthus cells, green dots) or not (black dots). Trend lines show local polynomial regression fitting and dark gray bands represent 95% confidence regions. The dataset for this figure and the R script used to analyze it and make the figure are available on Zenodo (10.5281/zenodo.10214013). (PDF) [file pbio.3002454.s005.pdf]

Figure S5.

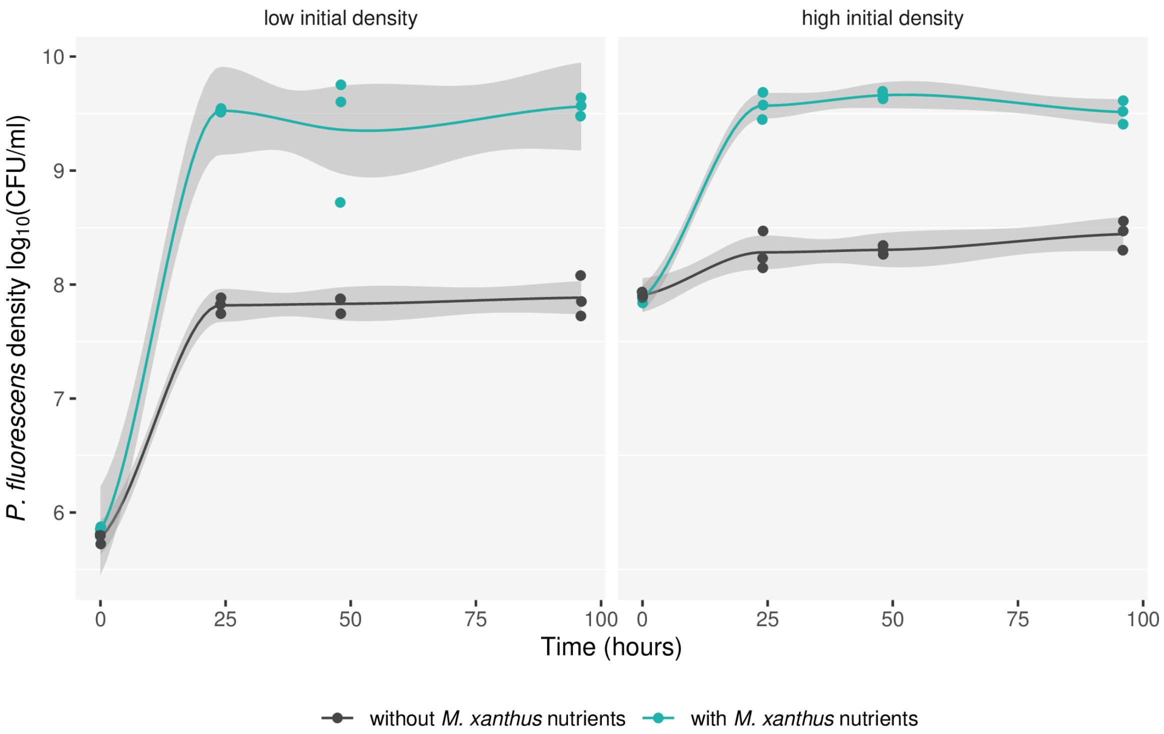

Reversed predation decomposes *M. xanthus* cells into diffusible nutrients. Remains of *M. xanthus* killed by 22 °C-reared *P. fluorescens* sufficient to fuel large *P. fluorescens* population growth pass through 0.2-µm filters within six hours of inter-species interaction. Estimated densities (log-transformed CFU/ml, $n = 3$) of *P. fluorescens* populations over time inoculated at two initial densities (~10^6 and ~10^8 CFU/ml) into supernatant from 22 °C-reared *P. fluorescens* lawns to which *M. xanthus* cells were either added (and which killed those *M. xanthus* cells, green dots) or not (black dots). Trend lines show local polynomial regression fitting and dark-gray bands represent 95% confidence regions. The dataset for this figure and the R script used to analyze it and make the figure are available on Zenodo (10.5281/zenodo.10214013).
