# Supplementary material for: Killer prey: Ecology reverses bacterial predation
Source: PLoS Biol. 2024 Jan 23;22(1):e3002454. doi: 10.1371/journal.pbio.3002454 (PMC10805292; doi:10.1371/journal.pbio.3002454)
Supplement: S6 Fig — Picture of 20-μl aliquots of supernatants from liquid suspensions of P. fluorescens grown on M9cas agar at 32°C (left) and 22°C (center) and of M9 buffer control (right). The 3 replicates of this assay yielded visually indistinguishable results; 1 replicate is shown here. (PDF) [file pbio.3002454.s006.pdf]

Figure S6.

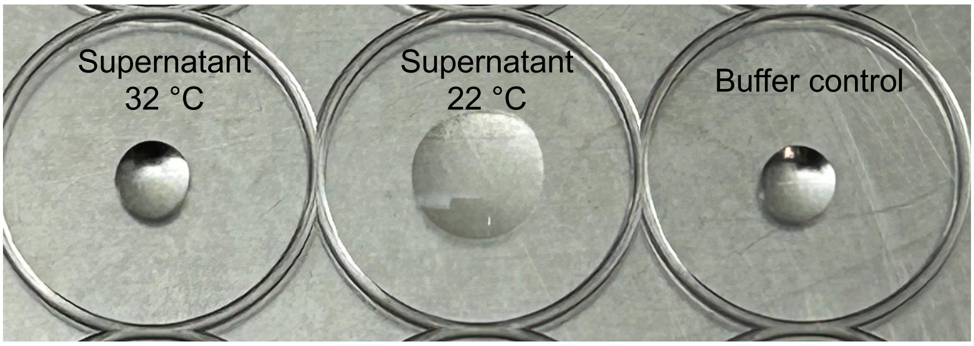

Drop-collapse assay with *P. fluorescens* supernatants. Picture of 20-µl aliquots of supernatants from liquid suspensions of *P. fluorescens* grown on M9cas agar at 32 °C (left) and 22 °C (center), and of M9 buffer control (right). The three replicates of this assay yielded visually indistinguishable results; one replicate is shown here.
