# Supplementary material for: Killer prey: Ecology reverses bacterial predation
Source: PLoS Biol. 2024 Jan 23;22(1):e3002454. doi: 10.1371/journal.pbio.3002454 (PMC10805292; doi:10.1371/journal.pbio.3002454)
Supplement: S1 Table — Linear model and Type III ANOVA for swarming data using prey identity, predator identity, temperature treatment, and their interactions as explanatory variables. Post hoc contrasts between temperature treatments are computed for each predator–prey combination. (PDF) [file pbio.3002454.s007.pdf]

Table S1. Statistical analysis of *M. xanthus* swarming data on prey lawns. Linear model and Type III ANOVA for swarming data using prey identity, predator identity, temperature treatment and their interactions as explanatory variables. Posthoc contrasts between temperature treatments are computed for each predator-prey combination.

A. Linear model

model <- lm(formula = swarm_diameter ~ prey * predator * temperature)

Multiple R-squared: 0.9325, Adjusted R-squared: 0.908
F-statistic: 38.08 on 62 and 171 DF, p-value: < 2.2e-16

Anova Table (Type III tests)
Response: swarm_diameter

	**Sum Sq**	**Degree of freedom**	**F value**	**Pr(>F)**
(Intercept)	52.926	1	442.719	< 2.2e-16 ***
prey	22.681	6	31.621	< 2.2e-16 ***
predator	8.065	2	33.733	4.478e-13 ***
temperature	0.035	2	0.146	0.864
prey:predator	10.384	12	7.238	1.234e-10 ***
prey:temperature	7.294	12	5.084	3.151e-07 ***
predator:temperature	0.022	4	0.047	0.996
prey:predator:temperature	1.439	24	0.501	0.975
Residuals	20.442	171		

Signif. codes: 0 = ***, 0.001 = **, 0.01 = *, 0.05 = .

B. Tukey-adjusted contrasts on temperature treatment for each predator-prey combination

pairs(emmeans::emmeans(model, "temperature", by = c("prey", "predator")))

```
prey = NO, predator = GJV1:
 contrast   estimate  SE  df.    t.ratio p.value
 12 - 22    0.1250 0.244 171   0.511  0.8660
 12 - 32    0.1000 0.244 171   0.409  0.9120
 22 - 32   -0.0250 0.244 171  -0.102  0.9943

prey = AG, predator = GJV1:
 contrast   estimate  SE  df.    t.ratio p.value
 12 - 22    0.1625 0.244 171   0.665  0.7843
 12 - 32    0.2875 0.244 171   1.176  0.4691
 22 - 32    0.1250 0.244 171   0.511  0.8660

prey = BB, predator = GJV1:
 contrast   estimate  SE  df.    t.ratio p.value
 12 - 22   -0.1250 0.244 171  -0.511  0.8660
 12 - 32   -0.0125 0.244 171  -0.051  0.9986
 22 - 32    0.1125 0.244 171   0.460  0.8899

prey = EC, predator = GJV1:
 contrast   estimate  SE  df.    t.ratio p.value
 12 - 22   -0.2250 0.244 171  -0.920  0.6282
 12 - 32   -0.3750 0.244 171  -1.534  0.2777
 22 - 32   -0.1500 0.244 171  -0.614  0.8129
```

prey = ML, predator = GJV1:

contrast	estimate	SE	df.	t.ratio	p.value
12 - 22	-0.2875	0.244	171	-1.176	0.4691
12 - 32	-0.0750	0.244	171	-0.307	0.9495
22 - 32	0.2125	0.244	171	0.869	0.6604

prey = PF, predator = GJV1:

contrast	estimate	SE	df.	t.ratio	p.value
12 - 22	0.7250	0.244	171	2.965	0.0096
12 - 32	-1.1875	0.244	171	-4.857	<.0001
22 - 32	-1.9125	0.244	171	-7.823	<.0001

prey = RV, predator = GJV1:

contrast	estimate	SE	df.	t.ratio	p.value
12 - 22	0.0250	0.346	171	0.072	0.9971
12 - 32	0.0750	0.346	171	0.217	0.9744
22 - 32	0.0500	0.346	171	0.145	0.9885

prey = NO, predator = A75:

contrast	estimate	SE	df.	t.ratio	p.value
12 - 22	0.0000	0.244	171	0.000	1.0000
12 - 32	0.0875	0.244	171	0.358	0.9319
22 - 32	0.0875	0.244	171	0.358	0.9319

prey = AG, predator = A75:

contrast	estimate	SE	df.	t.ratio	p.value
12 - 22	0.2250	0.244	171	0.920	0.6282
12 - 32	0.4125	0.244	171	1.687	0.2130
22 - 32	0.1875	0.244	171	0.767	0.7238

prey = BB, predator = A75:

contrast	estimate	SE	df.	t.ratio	p.value
12 - 22	0.0875	0.244	171	0.358	0.9319
12 - 32	0.0625	0.244	171	0.256	0.9646
22 - 32	-0.0250	0.244	171	-0.102	0.9943

prey = EC, predator = A75:

contrast	estimate	SE	df.	t.ratio	p.value
12 - 22	-0.1375	0.244	171	-0.562	0.8402
12 - 32	-0.1375	0.244	171	-0.562	0.8402
22 - 32	0.0000	0.244	171	0.000	1.0000

prey = ML, predator = A75:

contrast	estimate	SE	df.	t.ratio	p.value
12 - 22	0.2000	0.244	171	0.818	0.6924
12 - 32	0.0625	0.244	171	0.256	0.9646
22 - 32	-0.1375	0.244	171	-0.562	0.8402

prey = PF, predator = A75:

contrast	estimate	SE	df.	t.ratio	p.value
12 - 22	0.5125	0.244	171	2.096	0.0936
12 - 32	-1.8625	0.244	171	-7.618	<.0001
22 - 32	-2.3750	0.244	171	-9.714	<.0001

prey = RV, predator = A75:

contrast	estimate	SE	df.	t.ratio	p.value
12 - 22	0.0250	0.346	171	0.072	0.9971
12 - 32	-0.0250	0.346	171	-0.072	0.9971
22 - 32	-0.0500	0.346	171	-0.145	0.9885

prey = NO, predator = SO1:

contrast	estimate	SE	df.	t.ratio	p.value
12 - 22	0.1125	0.244	171	0.460	0.8899
12 - 32	0.1125	0.244	171	0.460	0.8899
22 - 32	0.0000	0.244	171	0.000	1.0000

prey = AG, predator = SO1:

contrast	estimate	SE	df.	t.ratio	p.value
12 - 22	0.1375	0.244	171	0.562	0.8402
12 - 32	0.2750	0.244	171	1.125	0.5001
22 - 32	0.1375	0.244	171	0.562	0.8402

prey = BB, predator = SO1:

contrast	estimate	SE	df.	t.ratio	p.value
12 - 22	0.1500	0.244	171	0.614	0.8129
12 - 32	0.1625	0.244	171	0.665	0.7843
22 - 32	0.0125	0.244	171	0.051	0.9986

prey = EC, predator = SO1:

contrast	estimate	SE	df.	t.ratio	p.value
12 - 22	0.0000	0.244	171	0.000	1.0000
12 - 32	-0.1000	0.244	171	-0.409	0.9120
22 - 32	-0.1000	0.244	171	-0.409	0.9120

prey = ML, predator = SO1:

contrast	estimate	SE	df.	t.ratio	p.value
12 - 22	0.0125	0.244	171	0.051	0.9986
12 - 32	0.0625	0.244	171	0.256	0.9646
22 - 32	0.0500	0.244	171	0.205	0.9772

prey = PF, predator = SO1:

contrast	estimate	SE	df.	t.ratio	p.value
12 - 22	0.5625	0.244	171	2.301	0.0584
12 - 32	-0.9375	0.244	171	-3.835	0.0005
22 - 32	-1.5000	0.244	171	-6.135	<.0001

prey = RV, predator = SO1:

contrast	estimate	SE	df.	t.ratio	p.value
12 - 22	0.2250	0.346	171	0.651	0.7922
12 - 32	0.1000	0.346	171	0.289	0.9549
22 - 32	-0.1250	0.346	171	-0.362	0.9305

P value adjustment: tukey method for comparing a family of 3 estimates
