# Supplementary material for: Killer prey: Ecology reverses bacterial predation
Source: PLoS Biol. 2024 Jan 23;22(1):e3002454. doi: 10.1371/journal.pbio.3002454 (PMC10805292; doi:10.1371/journal.pbio.3002454)
Supplement: S2 Table — Linear model and Type III ANOVA for M. xanthus growth data using P. fluorescens inoculum size, temperature treatment, their interaction, and time as explanatory variables. Post hoc contrasts between inoculum sizes are computed for each temperature–time combination. (PDF) [file pbio.3002454.s008.pdf]

Table S2. Statistical analysis of *M. xanthus* growth data. Linear model and Type III ANOVA for *M. xanthus* growth data using *P. fluorescens* inoculum size, temperature treatment, their interaction and time as explanatory variables. Posthoc contrasts between inoculum sizes are computed for each temperature-time combination.

A. Linear model

model <- lm(log10(predator_number +1) ~ prey_inoculum_size * rearing_temperature + time)

Multiple R-squared: 0.9407, Adjusted R-squared: 0.9286
F-statistic: 77.93 on 12 and 59 DF, p-value: < 2.2e-16

Anova Table (Type III tests)
Response: log10(predator_number +1)

	**Sum Sq**	**Degree of freedom**	**F value**	**Pr(>F)**
(Intercept)	244.066	1	315.709	< 2.2e-16 ***
prey_inoculum_size	192.800	3	83.131	< 2.2e-16 ***
rearing_temperature	0.049	2	0.032	0.969
time	10.421	1	13.480	0.001
prey_inoculum_size:rearing_temperature	195.261	6	42.096	< 2.2e-16 ***
Residuals	45.611	59		

Signif. codes: 0 = ***, 0.001 = **, 0.01 = *, 0.05 = .

B. Tukey-adjusted contrasts on prey inoculum size for each temperature-time combination

pairs(emmeans::emmeans(model, "prey_inoculum_size", by = c("rearing_temperature", "time")))

temperature = 12, time = 30 min:

contrast	estimate	SE	df.	t.ratio	p.value
no prey - 5	0.8001	0.508	59	1.576	0.3999
no prey - 6	0.7414	0.508	59	1.461	0.4675
no prey - 7	7.0188	0.508	59	13.826	<.0001
5 - 6	-0.0587	0.508	59	-0.116	0.9994
5 - 7	6.2187	0.508	59	12.250	<.0001
6 - 7	6.2773	0.508	59	12.366	<.0001

temperature = 22, time = 30 min:

contrast	estimate	SE	df.	t.ratio	p.value
no prey - 5	6.9464	0.508	59	13.684	<.0001
no prey - 6	6.9464	0.508	59	13.684	<.0001
no prey - 7	6.9464	0.508	59	13.684	<.0001
5 - 6	0.0000	0.508	59	0.000	1.0000
5 - 7	0.0000	0.508	59	0.000	1.0000
6 - 7	0.0000	0.508	59	0.000	1.0000

temperature = 32, time = 30 min:

contrast	estimate	SE	df.	t.ratio	p.value
no prey - 5	0.4443	0.508	59	0.875	0.8176
no prey - 6	0.4176	0.508	59	0.823	0.8435
no prey - 7	0.6910	0.508	59	1.361	0.5284
5 - 6	-0.0267	0.508	59	-0.053	0.9999
5 - 7	0.2467	0.508	59	0.486	0.9619
6 - 7	0.2734	0.508	59	0.539	0.9492

temperature = 12, time = 7 days:

contrast	estimate	SE	df.	t.ratio	p.value
no prey - 5	0.8001	0.508	59	1.576	0.3999
no prey - 6	0.7414	0.508	59	1.461	0.4675
no prey - 7	7.0188	0.508	59	13.826	<.0001
5 - 6	-0.0587	0.508	59	-0.116	0.9994
5 - 7	6.2187	0.508	59	12.250	<.0001
6 - 7	6.2773	0.508	59	12.366	<.0001

temperature = 22, time = 7 days:

contrast	estimate	SE	df.	t.ratio	p.value
no prey - 5	6.9464	0.508	59	13.684	<.0001
no prey - 6	6.9464	0.508	59	13.684	<.0001
no prey - 7	6.9464	0.508	59	13.684	<.0001
5 - 6	0.0000	0.508	59	0.000	1.0000
5 - 7	0.0000	0.508	59	0.000	1.0000
6 - 7	0.0000	0.508	59	0.000	1.0000

temperature = 32, time = 7 days:

contrast	estimate	SE	df.	t.ratio	p.value
no prey - 5	0.4443	0.508	59	0.875	0.8176
no prey - 6	0.4176	0.508	59	0.823	0.8435
no prey - 7	0.6910	0.508	59	1.361	0.5284
5 - 6	-0.0267	0.508	59	-0.053	0.9999
5 - 7	0.2467	0.508	59	0.486	0.9619
6 - 7	0.2734	0.508	59	0.539	0.9492

P value adjustment: tukey method for comparing a family of 4 estimates
