# Supplementary material for: Killer prey: Ecology reverses bacterial predation
Source: PLoS Biol. 2024 Jan 23;22(1):e3002454. doi: 10.1371/journal.pbio.3002454 (PMC10805292; doi:10.1371/journal.pbio.3002454)
Supplement: Attachment — Submitted filename: Response_document.pdf [file pbio.3002454.s009.pdf]

As you will see below, the reviewers find the work interesting, but they all have concerns that will need to be solved before further consideration. We think that it is important that you include novel experiments in which the predation assays with predator & prey grown at the same temperatures (ie, pre-incubation of both species at the same temperature as the predation assays are performed at), and include an experiment in which killing by & growth of P. fluorescens is done in the same experiment, i.e. currently, you show killing by P. fluorescens in one experiment and then in a different experiment show that P. fluorescens can grow on the supernatant from the lysed Myxococcus cells. We also think that either including the data for the drop collapse experiment mentioned in the Discussion or not mention this experiment at all, and including more information from the literature about the antimicrobial activity of P. fluorescent would be important. Please also address the other reviewers' points by editing the text/title.

We thank the three reviewers for their thoughtful reading of and comments on our manuscript, which have helped us improve it. As requested, we now present i) an experiment in which both species were both pre-incubated at the same temperature as used in the interaction assays (for both 22 and 32 °C), ii) an experiment in which killing by *P. fluorescens* & growth of *P. fluorescens* on *M. xanthus* remains occurs in the same experiment, and iii) data for the drop-collapse experiment mentioned in the Discussion. We additionally include more information from the literature about the antimicrobial activity of *P. fluorescens*. We respond to the reviewers' comments in detail below, pointing out corresponding changes to the manuscript. New or revised text in the manuscript is identified with blue text.

Reviewer #1: *The manuscript by Vasse et al. describes a study of predation between Pseudomonas fluorescens and Myxococcus xanthus, which changes sign depending on what temperature the P. fluorescens was reared. The authors show that cold-grown P. fluorescens produces a diffusible non-proteinaceous compound which kills M. xanthus, and P. fluorescens is able to grow on the nutrients released by killed M. xanthus.*

The manuscript is well-written, the experiments are described clearly and the conclusions are supported by the data.

Did the authors do predation assays with M. xanthus and P. fluorescens at the three different temperatures? ie with pre-incubation of both species at the same temperature the predation assays were then performed at? It seemed strange to start with a setup where pre-incubation temperatures were varied and the predation assays temperatures weren't.

Similarly, does P. fluorescens still prey upon M. xanthus at 22 oC rather than the other way round, if both organisms were pre-incubated at 32 oC.

In our first submission, we had manipulated only the pre-interaction growth temperature of *P. fluorescens,* whereas all predation assays were performed at 32 °C; the motivation for this approach is mentioned in the last paragraph of the Introduction, where we also define the central theme of the paper as "how variation in the temperature at which bacterial prey grow prior to predator attack might alter the predation risk of prey".

However, because the reviewer and editor expressed interest in outcomes when pre-incubation and predation occurred at the same temperature, we performed a new experiment with two temperature regimes, one in which pre-incubation and species interaction both occurred at 32 °C – a treatment that was in fact already part of our previous experimental design – and a second treatment in which pre-incubation and species interaction both occurred at 22 °C. As expected, *M. xanthus* killed *P. fluorescens* when both were pre-grown and interacted at 32 °C whereas *P. fluorescens* killed *M. xanthus* when both were pre-grown and interacted at 22 °C. We show these results in a new supplemental figure (Fig. S4).

How long does the pre-incubation acclimation last?

The pre-incubation acclimation at room temperature (after initial prey growth and before predator-prey interaction) lasts for two hours.

Specific Comments

I found the title a bit difficult to get my head around, and only really understood what the authors meant after reading the abstract. It might be worth considering a longer but clearer title.

We propose a slightly modified title that we hope will be a bit easier to grasp at first reading: "Killer prey: Pre-interaction ecology reverses bacterial predation".

Line 120. Spell out rH as relative humidity

We spelled out rH as suggested.

Line 149. It wasn't clear until quite a lot later why flasks were being used rather than petri dishes. Might make sense to explain the rationale earlier?

We now explain the rationale for using flasks rather than Petri dishes when flask usage is first mentioned in the Methods.

Line 226. To harvest P. fluorescens supernatant, ...

This step is actually used to harvest *P. fluorescens* and the supernatant is isolated by centrifugation in a subsequent step. We have rephrased to make this clearer.

Lines 251-4. To what extent does the pre-growth of prey lawns reduce the concentration of free nutrients for M. xanthus growth? These few lines seem a bit vague.

P. fluorescens cells have the opportunity to grow on the casitone initially present in the agar medium for a full 24 hours before we introduce *M. xanthus*. While we have not quantified the level of casitone remaining in the agar after that period that might be available to *M. xanthus* for growth, we expect that it is very low. This is because P. fluorescens grows to similarly high densities as *M. xanthus* in liquid cultures in which casitone is the sole carbon source. We have slightly revised this section for clarity.

Line 258 'addition' might be better than 'release'.

We prefer "release" over "addition" in the description of the predation experiment by the motile bacterium *M. xanthus.*

Reviewer #2: *Authors present an interesting study with the main finding of temperature-dependent predation evasion of Myxococcus xanthus by Pseudomonas fluorescens. The authors also make a claim of inversion of predator-prey relationship at the temperature that P. fluorescens kills M. xanthus, however the evidence presented is not strong enough for how emphatic these claims are. So while the overall study is interesting and should be published, the claims of predation inversion need to be reduced considerably. The discussion is especially problematic and must be remedied prior to publication.*

Otherwise, the paper is reasonably clearly written and the figures support all other conclusions well. The discussion hinges strongly on the predation reversal claim, of which the evidence presented is not strong enough to warrant this level of certainty.

We consider the inference of predation reversal based on the multi-step experiment presented in our original submission to be justified, and indeed very strong. Nonetheless, we thank the reviewer for their comments on this point, as they prompted us to perform a new experiment that we think nicely supplements our original experiment and further strengthens the inference of predation reversal. In this experiment, described in greater detail below, we demonstrate that the *same culture of* 22C-grown *P. fluorescens* both kills added *M. xanthus* and fuels growth from nutrients derived from the killed *M. xanthus* cells. In contrast, 32C-grown *P. fluorescens* populations are greatly reduced by addition of *M. xanthus.*

In the discussion it is also claimed that the killing molecules (no evidence whether it is one or multiple compounds mediating the killing) is "extremely effective", however no serial dilution experiment was done, just the 30 minute exposure experiment with no earlier time points. This speed of killing is quite fast, but is not used to follow up on the possible nature of the killing substance.

By "extremely effective", we are simply referring to the fact that *M. xanthus* populations are completely (or nearly completely) killed when exposed to supernatant from 22C-grown *P. fluorescens* within only 30 minutes. Although we think that was fine, we have changed 'extremely' to 'highly'.

Additionally, in the discussion a result about a drop-collapse experiment is mentioned, but these data are not included in the results section.

We include a new supplemental figure (Fig. S6) showing an image of the supernatant aliquots examined in the drop-collapse experiment.

In general, the discussion section is too expansive and speculative. It should be shortened substantially and edited to focus on the results presented. For example, the section about 'past ecology shaping future interactions': no co-culture experiments were presented which could have tested whether temperature also determined the nature of the interaction when the two species are grown together, instead the results are extensively re-described and speculated upon.

In this section, we discuss how which species acts as predator and which as prey is determined by the temperature at which *P. fluorescens* grew prior to the interaction with *M. xanthus* (hence in the past). We believe that this observation is particularly interesting because (i) while the importance of the ecological context in which an interaction occurs is widely acknowledged, prior ecological effects are rarely considered, and (ii) it contributes to the challenge of predicting interaction outcomes. We therefore believe that this section is useful in the Discussion.

We now share our perspectives regarding the more stylistic points on brevity, re-description and speculation. Concerning re-describing results, we consider such re-description in a Discussion section, ideally in a new form, to often be an important strategy for reinforcing understanding and reminding readers of the outcomes most directly relevant to the points being discussed. With respect to speculation, we consider plausible speculation to be an important exercise for promoting reflection on both clear and potential implications of experimental results. It is thus also important for stimulating thinking about how those results might inform future research, whether by the authors or readers. Indeed, we consider plausible speculation about potential implications to be one of the most important elements of a thought-provoking and hence productive Discussion.

Additionally, many statements in the discussion are presented without appropriate references. For example: L460 "Within such communities, micro-ecological features can change rapidly over time and migration across variable microenvironments can be extensive."

By saying that "Within [natural microbial] communities, micro-ecological features can change rapidly over time and migration across variable microenvironments can be extensive.", we simply meant that microbial communities in nature can experience rapid changes in their environmental conditions, for example due soil-surface-temperature changes between day and night and sudden moistening of dry soil due to rain, and also that cells in such communities can easily be translocated to a different environment, for example due to water flow, animal movements or their own motility. Beyond this statement, we did not find any statements in the Discussion requiring a reference that lack one.

Specific Comments

Predator genotype, prey identity and temperature affect swarm diameter, and interaction. While the effect of P. fluorescens is dramatic, the other effects are minor and should be presented as such.

After describing the general effects, we explain each of them in greater detail and state that only the effect of *P. fluorescens* is substantial. In addition, all related statistical tests and their outputs are available as supplementary material.

The reduction of swarming radius when prey is present vs not, implies that a carbon free medium that forces the predator to consume prey to swarm at all may have been a better control, as the current implication is that prey presences reduces swarming.

In retrospect, we agree that a control treatment with neither casitone nor prey would have been nice to have included as a baseline comparison as well, but its absence doesn't compromise any of our inferences. Prey do indeed generally decrease the rate at which *M. xanthus* can swarm relative to agar-only media in which all available carbon-substrate is in the form of casitone. It is likely harder for *M. xanthus* to extract nutrients from prey cells than directly from agar medium, and prey lawns might also be less conducive to fast swarming than agar alone. However, our primary comparisons of interest are across prey treatments and temperature treatments.

This sentence is very confusing and should be re written to be more understandable. "We hypothesized that, in the Fig. 2 experiment with DK3470, population densities of P. fluorescens after overnight growth might have mediated some effects of P. fluorescens on DK3470 growth or death, whether partially or fully."

We have rewritten this sentence to read: "We hypothesized that, in the Fig. 2 experiment with DK3470, differences in population densities of *P. fluorescens* across treatments might have been at least partially responsible for differences in how *P. fluorescens* grown at different temperatures interacted with DK3470."

Did you check the pH of the P. fluorescens supernatants from different temperatures? If the pH is the same then this would support the killing of M. xanthus being caused by a more interesting molecule. The speed of killing implies this could be pH mediated.

We thank the reviewer for this interesting question. As part of our new experiment reported in the new Fig. 4, we measured the pH of supernatant from resuspended 22C- and 32C-grown cultures of *P. fluorescens* and found no difference. We now report this absence of effect in our revised manuscript and discuss its implications.

Just because P. fluorescens can grow on the supernatant from killed M. xanthus, doesn't mean that the killing by P. fluorescens is predatory. This evidence is not strong enough to say that the direction of predation is temperature dependent. If it could be shown that P. fluorescens kills and eats M. xanthus in the absence of any other nutrient source at 22C, then this would be definitive evidence that the direction of predation is changed. However this experiment needs to be done with both species co-cultured, and to truly be definitive it would be nice to see radionuclide labeled M. xanthus incorporated into P. fluorescens, though this is extremely high level of evidence. Co-culturing without exogenous nutrients and P. fluorescens growing better would be enough to support predation.

While we do think that our previous experiment gave strong evidence of temperature-dependent predation reversal, we agree that showing that a single culture of 22C-grown *P. fluorescens* that kills *M. xanthus* and then also grows on nutrients derived from the killed *M. xanthus* cells is ideal. We provide a new experiment that shows this.

In this new experiment, we added samples of *M. xanthus* culture covering the entire surface of small lawns of *P. fluorescens* grown at 22 or 32 °C. We sought to add enough *M. xanthus* cells so that the nutrients from cells killed by 22C-grown *P. fluorescens* would suffice to fuel extensive and readily detectable growth of *P. fluorescens* beyond the growth it could achieve in the absence of *M. xanthus*. We were successful with this approach, showing that small lawns of 22C-grown *P. fluorescens* populations to which *M. xanthus* cells were added not only completely killed *M. xanthus* at our level of detection (as seen in our prior experiments), but then subsequently grew to a population size about two-fold larger, on average), than populations to which *M. xanthus* was not added. Neither effect was seen for 32C-grown *P. fluorescens* populations, which were reduced by ~ 87% on average by addition of the same number of *M. xanthus* cells.

Rationale for our original experimental design: We originally took a two-culture-stage approach to avoid potential difficulties of having relative *M. xanthus vs P. fluorescens* population sizes in co-cultures that might not allow clear detection of *P. fluorescens* increase fuelled by *M. xanthus*. For example, adding few *M. xanthus* cells relative to 22C-grown *P. fluorescens* cells would still allow detection of *M. xanthus* death but might not provide enough detritus from killed *M. xanthus* cells to fuel detectable growth by the *P. fluorescens* population. Inversely, adding

far more *M. xanthus* than *P. fluorescens* cells might reduce what percentage of *M. xanthus* cells are killed by 22C-grown *P. fluorescens*.

Indeed, in our original experiments the *P. fluorescens* population grown up on plates was much greater in size than the *M. xanthus* populations that we added and were killed, because those *M. xanthus* samples were added to just a small center portion of the *P. fluorescens* lawns. Thus, the nutrients from the killed *M. xanthus* cells would likely not have been sufficient in quantity to drive detectable growth by the much larger *P. fluorescens* population. In our new experiment, we solved this by adjusting the size of the *P. fluorescens* lawn and the population sizes of the added *M. xanthus* samples. We succeeded at finding an *M. xanthus* population size that both was thoroughly killed when added to 22C-grown *P. fluorescens* and that fueled subsequent growth by the same *P. fluorescens* culture.

However, we think our original experiment remains valuable alongside the new one and we thus opt to keep it in the manuscript, but now as a supplemental figure. That original experiment shows not only that *M. xanthus* cells killed by 22C-grown *P. fluorescens* can fuel extensive subsequent growth by *P. fluorescens*, but also that the nutrients from the killed *M. xanthus* cells rapidly become highly diffusible, passing through a 0.2-micron membrane, an observation with implications for understanding the spatio-temporal accessibility of nutrients from killed *M. xanthus*.

The observation that P. fluorescens supernatant is only lethal to M. xanthus is very interesting. It would also be interesting to know if it can kill eukaryotic predators like Tetrahymena or Acanthamoeba.

We agree that a deeper exploration of the supernatant killing range would be very interesting, but we consider this to be beyond the scope of the present study.

There is a section of the discussion about a drop-collapse experiment to show that the killing substance is a surfactant, but these data are not in the results. Either this section needs to be removed or the data presented.

We now show a picture of the supernatant aliquots examined in the drop-collapse experiment as a new supplemental figure (Fig. S6).

This sentence (Line 454-458) is misleading. "Yet differences in P. fluorescens biotic environments caused by the differences in growth temperature were not eliminated by the subsequent temperature equalization. P. fluorescens reared at 32 °C was extensively killed when exposed to M. xanthus (Fig. S1), whereas P. fluorescens reared at 22 °C slaughtered M. xanthus to extinction (Figs. 2, 3 and S2) and used the released nutrients to fuel its own growth (Fig. 4)." This implies that the evidence for P. fluorescens "fueling its own growth" on killed M. xanthus comes from the same experiment as the killing of M. xanthus, which is not the case. This needs to be fixed before publication as it greatly overstates the evidence presented.

We were transparent with our phrasing that our understanding that 22C-grown *P. fluorescens* was consuming dead *M. xanthus* in the original plate experiments was an inference from results of the Fig. 4 experiment. While we considered this inference to be strongly justified (and still do), we now provide evidence of *P. fluorescens* predation on *M. xanthus* from a single-culture experiment, as described above.

Other problematic statements: L468 "The finding that some bacteria can be predators of myxobacteria". The evidence presented isn't sufficient to claim a single predator of M. xanthus, nevermind some predators of myxobacteria in general.

We have changed this sentence to read: "The finding that a non-myxobacterial species can be a predator of *M. xanthus rather* (…)".

This section adds very little of interest: "The ecological dependence of microbial predator-prey interactomes and antagonisms more broadly also has applied significance (63-65). The range of ecological conditions under which microbial biocontrol agents can be expected to harm their targets should be investigated in planning their use."

We consider this point worth making, even if not all readers find it interesting.

"How much microbial warfare results in predation?". This section is generally problematic. The evidence presented in this paper does not show that P. fluorescens is preying upon the M. xanthus, since the way the experiment was done was highly artificial. It doesn't even fit the authors definition since the P. fluorescens that killed the M. xanthus are not the same cells that consumed the nutrients, so technically this would be scavenging..

Please see our response to the similar comment above. Consistent with our prior experiments, our new experiment shows that the same 22C-grown *P. fluorescens* cultures that kill *M. xanthus* also grow specifically due to the presence of the *M. xanthus* cells that they kill.

The next paragraph then also remains highly speculative: far more evidence is required to show that other bacteria that may incidentally consume nutrients derived from killed competitors are predators. Myxobacteria and BALOs are considered specialized bacterial predators due to large bodies of evidence, such evidence is not even close to existing for other bacteria.

By the definition of predation we adopt, which is a common one and does not include adaptive criteria such as being "specialized", even "incidental" consumption of killed-cell nutrients by the killer intrinsically constitutes predation. In the second paragraph of this subsection we speculate intentionally, and we think plausibly; it is our intent to be thought-provoking with this paragraph.

Reviewer #3: *Comments to the authors*

In their manscript, Vasse et al. describe the influence of an abiotic factor, i.e. temperature, to antagonistic interbacterial interactions. Specifically, they focus on the predator-prey relationship of the predatory bacterium Myxococcus xanthus with Pseudomonas fluorescence. From several quantitative co-culture experiments the authors deduce that P. fluorescence reared at 32C serves as prey for M. xanthus, while P. fluorescens reared at lower temperature (22C) acts as a predator on M. xanthus. Further experiments with P. fluorescens culture supernatant suggests that not-proteinaceous diffusible molecules are effective in killing M. xanthus.

The experiments are sound, data are well presented and evaluated, which includes appropriate statistics. The significance of the observations are discussed in the broader context of the ecological relevance of interspecies interactions and how they are shaped by environmental changes.

The findings are very interesting and will promote new thoughts on our understanding of bacterial antagonistic interactions, specifically predation. Especially the observation that P. fluorescence grows on nutrients from M. xanthus alone challenges our definition of bacterial predation, which might be a much more common strategy than currently recognized.

My main comment is that the study should relate more to known literature. Antimicrobial activity of Pseudomonas fluorescence has been demonstrated before, and the species as been described as a biocontrol agent promoting plant growth by inhibiting (fungal) plant pathogens (see for example the review Haas, D., Défago, G. Nat Rev Microbiol 3, 307-319 (2005).; also, there are lots of studies on the interaction with e.g. Ralstonia sp.).

It is also known that P. fluorescence produces siderophores and antibiotics, but also volatile toxic compounds, such as cyanide, which should be discussed in the context of the presented observations. Even more, it was shown that cyanide production depends on growth phase of P. fluorescence (e.g. Askeland RA, Morrison SM Appl Environ Microbiol. 1983)

So, the question arises whether the observed temperature-dependent effect of P. fluorescence may really show a dependence on growth phase, as temperature obviously affects growth rate. This is could be relevant not only in the context of cyanide, but also of other antimicrobial agents.

I believe the authors could come up with an experiment to test this - I consider it important, as it might alter the way they discuss their observations.

We now address previous literature related to anti-microbial effects of *P. fluorescens* more extensively, particularly at lines 571-575. Regarding the cyanide/growth-phase hypothesis, we appreciate and share the reviewer's interest in the molecular mechanism behind this killing effect. However, we have chosen to focus on the population-biology aspects of predation reversal in the current manuscript, which we think are highly novel and significant in their own right. While growth-phase dependence would be interesting to test from a mechanistic perspective, we don't feel this point bears greatly on any of the major points we wish to make with this current manuscript and thus would not opt to pursue this currently.

Specific comments:

- p. 7, l. 4: "LB 0.5% agar". Does 0.5% refer to NaCl concentration, or agar concentration (soft agar)?

It refers to the agar concentration; we now add a mention of soft agar.

- p. 17, l. 422-423: The authors mention a "drop-collapse assay" suggesting the antimicrobial compound is a biosufactant. Is the experiment included in the manuscript or not? If these are preliminary observations it should please be stated as such, and "data not shown"

As noted above, we include a new supplemental figure (Fig. S6) showing an image of the supernatant aliquots examined in the drop-collapse experiment.

- p. 11, l. 261: "across the dataset, swarm sizes were impacted by [.....] the rearing temperature." It looks to me that only for P. fluorescence has an impact?

The effect of prey identity differs depending on the rearing temperature (prey:temperature interaction, $F_{12, 171}$ = 5.084, $p < 0.01$, Table S1A). This means that at least one combination of prey and temperature is different from the others (effect of *P. fluorescens*, Table S1B). We realized that the two supplementary tables (presenting the statistical tests) were somehow trimmed and this seen incompletely in the submitted version of our manuscript, we now replace them by the complete tables.
