# Supplementary material for: Killer prey: Ecology reverses bacterial predation
Source: PLoS Biol. 2024 Jan 23;22(1):e3002454. doi: 10.1371/journal.pbio.3002454 (PMC10805292; doi:10.1371/journal.pbio.3002454)
Supplement: Attachment — Submitted filename: Response document.pdf [file pbio.3002454.s010.pdf]

Response document

Thank you for your patience while we considered your revised manuscript "Killer prey: Pre-interaction ecology reverses bacterial predation" for consideration as a Short Reports at PLOS Biology. Your revised study has now been evaluated by the PLOS Biology editors, the Academic Editor, and two of the original reviewers.

In light of the reviews, which you will find at the end of this email, we are pleased to offer you the opportunity to address the remaining points from the reviewers in a revision that we anticipate should not take you very long. We will then assess your revised manuscript and your response to the reviewers' comments with our Academic Editor aiming to avoid further rounds of peer-review, although might need to consult with the reviewers, depending on the nature of the revisions.

In particular, we think that the additional experiments mentioned by reviewer #2 are not necessary for publication. We think it is important that you address the points raised by reviewer #3 and that you clarify/edit the text to address the criticisms raised by reviewer #2.

We thank both reviewers for their further thoughtful comments and the editors for their positive assessment and clear guidance. Below we respond to the reviewer comments and indicate any changes to the manuscript made in response. In addition to the changes mentioned below, we have added one sentence to the Discussion to more specifically illustrate the likely implication of our results that more forms of microbial warfare result in predation than has previously been recognized.

REVIEWS:

Reviewer #2: This version is somewhat improved but I still think the evidence for predation inversion is insufficient. The authors clearly think the evidence is much stronger, so either this should be published to let readers decide or the editor can decide how strong the evidence presented is.

We do indeed think the evidence for predation inversion is strong, as commented on further below.

I also think that for a short report the discussion is far too long and strays away from discussing the results of the presented work into a perspective on antagonism and predation, which is a detraction from the work.

We hold a different view. We consider the length appropriate and request that the editors allow readers to judge the likely implications we draw from our results regarding lethal antagonisms traditionally understood and predation. We consider those implications to be among the most important take-aways

from the paper and we predict that this material will stimulate much fruitful discussion and new lines of future research in the microbial ecology community.

Why I think the evidence is insufficient:

1. Experiments showing that M. xanthus kills P. fluorescens at 32C (Fig S1) are clear, but accompanying evidence that predation is also occurring is insufficient. Figure 1 shows mostly a reduction in swarm diameters on lawns of other bacteria. References 18 and 40 appear to use agar without 0.3% casitone, so the evidence for predation is much better. It's unclear why 0.3% casitone plates were used in the present study. The response to my previous comment indicates that M. xanthus is indeed using the casitone for part of its growth which makes it harder to conclude the amount of predation it is engaging in. In Figure 4 the increase of M xanthus suffers from the same lack of controls as described below.

We have demonstrated growth of *M. xanthus* fueled by *P. fluorescens* grown at 32 °C in multiple previous studies. The purpose of Fig. 4b (right panel) is to compare the effects of *P. fluorescens* growth temperature on the temporal trajectory of *M. xanthus* population size after initiation of interaction, not to quantify the precise relative degree *M. xanthus* growth due to predation vs. any potential growth on residual casitone not previously consumed by *P. fluorescens* prior to addition of *M. xanthus*. Fig. 4b shows the fold-change in *M. xanthus* population size after 24 hours relative to the starting size, for both *P. fluorescens*-temperature treatments. On 32 °C-grown *P. fluorescens, M. xanthus* kills ~90% of *P. fluorescens* and increases greatly in population size, as expected. On 22 °C-grown *P. fluorescens, M. xanthus* populations decrease to zero within 24 hours. Given the death of 90% of *P. fluorescens* cells, we consider it highly implausible to suggest that *M. xanthus* growth is fueled 100% by residual casitone not previously used by *P. fluorescens* and 0% from consumption of nutrients derived from the 90% of *P. fluorescens* killed by *M. xanthus.*

2. Experiments showing that P. fluorescens kills M. xanthus at 22C are clear, but the evidence for predation is insufficient. As far as I can tell in the methods for the experiment presented in Figure 4, there is no control to show that the modest increase in P. fluorescens (~2-fold) after 24 h after addition of M. xanthus is due to predation.

Two controls are missing: one where nothing is added to the P. fluorescens lawn, and one where the same amount of M9 buffer the M. xanthus cells were resuspended in is added. Based on the text in the results maybe this was done? But in the methods it is not described.

The appropriate control was indeed performed and is referred to in the main text, as noted by the reviewer ("Importantly, 22 °C-reared P. fluorescens populations were additionally observed to act as predators of M. xanthus by growing ~80% more in size when *M. xanthus* was added than on M9cas alone"). However, the reviewer's comment made us realize that we did not explicitly refer to that control treatment in the Methods; we apologize for this and thank the reviewer for drawing this to our attention. Growth by *P. fluorescens* on M9cas agar alone - the control the reviewer inquires about - was assessed

and is intrinsic to Fig. 4a, being represented by the value of 1.0 on the y-axis. We now explicitly describe the control in Methods as well as referring to it in the figure legend. The control was performed by adding the same amount of M9 liquid buffer (with no casitone) that the *M. xanthus* cells were resuspended in. That control was identical to the other treatment except for the presence of *M. xanthus* cells in the M9 buffer. The other treatment suggested by the reviewer - *P. fluorescens* alone without the addition of buffer - is not necessary in our view.

Given the control, the evidence for predation is clear. There is no plausible explanation for the ~80% increase in *P. fluorescens* population size caused by the presence of *M. xanthus* - which *P. fluorescens* slaughters rapidly and completely - other than that *P. fluorescens* is growing on biomass from dead *M. xanthus* cells. Regarding the reference to a "modest" increase, we suggest that an 80% increase in predator population size is very large, not modest. We suspect that in thinking about animal predators, e.g. lions, if the presence of a prey species were shown to cause an 80% increase in a lion population over a short period, most would consider this increase to be large.

3. It is clear that the 22C supernatant from P. fluorescens lyses M. xanthus cells, providing nutrients for growth.

4. The discussion of the density-dependence of the killing factor production has issues: The logic that because the 22C grown cultures all had comparable density to the 32C cultures, but killing of M. xanthus only occurs at 22C, therefore density doesn't affect killing is undermined by the observation that density does affect killing at 12C. It seems likely that lower initial inocula at 22C would result in less growth and at some point, affect killing. So as currently written, the results do not support the conclusion. Either lower inocula densities at 22C need to be tested, or it should be concluded that killing of M. xanthus is density-dependent.

This is stated in the discussion, so the results section should be edited to make this conclusion clearer. The paragraph around Line 530 is predicated on the lack of experiments done at 22C with lower densities which would allow the conclusion that the killing molecule is density dependent, but not produced at 32C.

We do not agree that the referred-to paragraph is predicated on experiments that were not performed. In that paragraph we do not conclude that "*density doesn't affect killing*". After arguing that density does appear to affect killing at low temperatures (e.g. 12 °C), we only conclude that "high density alone is insufficient to allow predation of *M. xanthus* by *P. fluorescens*". This is because 22 °C- and 32 °C-reared *P. fluorescens* both reach similar high densities but the 32 °C-reared cells do not kill *M. xanthus.* Thus, high density *per se* is insufficient to generate killing. To improve clarity, we propose to add to the end of that sentence: "high density alone is insufficient to allow predation of *M. xanthus* by *P. fluorescens*; <u>temperature is also determinative</u>" (new text underlined).

In the Results, we do not state that our results demonstrate that density-dependence does not occur at 22 °C, but only that they fail to provide positive evidence for such. This is an important distinction. Moreover, we qualify our statement as being relevant only to the density ranges of our experiment. We write: “our results do not indicate that *P. fluorescens* post-growth population density has any effect on the efficacy with which 22 °C-grown *P. fluorescens* kills *M. xanthus,* at least over the range observed here”.

Further notes on the evidence for predation by P. fluorescens

In the methods section "Tests for growth of P. fluorescens on nutrients from M. xanthus killed by P. fluorescens and for effects of P. fluorescens rearing temperature on M. xanthus DK3470 and P. fluorescens population sizes after interaction", there does not appear to include a control where just 20uL M9 was added on top of the P. fluorescens lawns, or nothing. So, it's hard to conclude that the growth that occurred in the next 24h of the lawns where M. xanthus was added is indeed due to nutrients released from killing.

But in the results:

Line 438 "to act as predators of M. xanthus by growing ~80% more in size when M. xanthus was added than on M9cas alone". This does not match the methods description around line 240, nor the caption of Figure 4.

As addressed also above, we apologize that we did not describe the control referred to in the Results also in the Methods. The controls are now mentioned in the Method section and in the figure legend.

If M9 with cas was added this isn't a good control since the cas could be used for growth. The experiment should be M. xanthus cells in M9 salts, or just M9 salts, or nothing. Then compare the growth after 24h. If P. fluorescens grows more with M xanthus cells then it killed and ate them and predation is well supported.

The control was indeed just M9 salts liquid, not M9cas liquid, as also addressed above. We estimated the growth of *P. fluorescens* both in the presence of *M. xanthus* resuspended in M9 liquid (without any casitone) and, as a control, in the presence of the same volume of M9 liquid (with no cas). Given these controls and the 80% increase in *P. fluorescens* growth in the presence of *M. xanthus*, we agree with the reviewer that we can safely conclude that predation is well-supported.

Further notes about the discussion being too long

The discussion is very long and proscriptive, especially for a short report. The paragraph around line 540 could be greatly shortened.

We have carefully re-read our Discussion in light of the reviewer's comment and have concluded that the themes of the Discussion and the extent to which we address them seem appropriate. We strongly prefer to keep the Discussion in its current form unless the editors insist on changes as a condition of publication. We're quite surprised at the description of the discussion as proscriptive, which we understand to mean denunciatory or condemnatory; we don't think those terms accurately describe our discussion at all.

The paragraph around line 560 feels very strong given the weakness of the predation inversion evidence as described above.

We think the evidence for predation is strong, as outlined above.

The paragraph around line 570 veers into unrelated territory, as the presented study does not address antagonism, since the message is clearly about predation. Overall, this paragraph reads as a perspective, pushing the notion that interference competition frequently involves predation. This does not belong in a short report.

Like many others, we consider predation to be a sub-category within the broader category of antagonistic interactions. We consider drawing likely implications of the presented work to be a very important aspect of a discussion section and strongly disagree that this material is inappropriate in this context. We think our results strongly suggest that lethal microbial antagonisms not traditionally associated with predation in fact often mediate predation. If this proves to be the case, as we predict, it will represent a substantial shift in how such antagonisms are understood. We consider this prospect to be one of the most important and exciting aspects of this study.

Other issues in this paragraph:

"P. fluorescens has not often, if ever, been described as a predator". This should be answerable in the literature. I expect the answer is never.

We expect the same and have found no such instance in the literature, but we phrased this conservatively in case we missed something. We have changed this phrasing to: "it does not appear that *P. fluorescens* has previously been labeled a predator".

"because the strain used here can clearly grow on nutrients derived from cells it has killed". Again, this is not clear from the evidence presented. Figure 4 lacks controls.

The control needed to show *P. fluorescens* grows on killed *M. xanthus* was performed, as addressed above.

Other remarks

73 "Predatory weapons" this is confusing terminology. Weapons should be used to describe systems used for interference competition between peers, not predatory adaptations. As in ref 29.

We respectfully disagree with this opinion, seeing no reason to limit 'weapons' to agents of harm that never result in predation, especially since we suggest that many microbial weapons not traditionally associated with predation in the literature are so associated in reality. Both of the phrases 'predatory weapons' and 'weapon(s) for predation' are already established in the literature.

Line 303. Sequester is not the right word to use here. The lawn bacteria are using the casitone for anabolism, not just taking it up and holding on to it.

Sequestration is commonly used to refer to the transfer of nutrients from an abiotic environment into biomass.

Line 525. Instead of the review reference 61, the exact references for M. xanthus and Streptomyces secreting diffusible killing molecules and eating prey should be provided.

We now provide references for *M. xanthus* and *Streptomyces* secretion-mediated predation.

The supernatant should also be treated by proteinase K to affirm its non-proteinaceous nature. This is a pretty standard assay.

Given that *P. fluorescens* is not an extreme thermophile, we consider retention of function after experiencing 95-degree heat to be strong evidence that the focal molecule is not a protein.

Figure 4 Caption mentions lighter dots, but I only see the one dot with lines. Either the caption needs editing or the plot corrected.

I appreciate the inclusion of the drop-collapse data, but looking at Figure S6 I can't see any difference between the three plates. Either an arrow needs to be added or a picture from a different angle or with a black background or something because this is not clear at all.

Regarding these last two points, we apologize for issues in converting our word document into a pdf document. During the conversion, the figures got modified. We now provide the original versions of the figures, with the droplets for figure S6 and the lighter dots for figures 4 and 5.

—-------------------------------

Reviewer #3: I find that the authors have further improved their very interesting manuscript: they amended the text and added new experiments, or argued convincingly when they decided against addressing a specific point. I have only two minor points to be addressed, outlined below.

We thank the reviewer for their positive assessment of our revised work.

New Figure 4 and corresponding text: This new experiment nicely supports the main claims. However, the figure only shows the fold-change in population size but gives no information about the absolute population sizes, nor (in the methods section) on the number of M. xanthus cells added to P. fluorescence. To better interpret to what extent M. xanthus fuels growth of P. fluorescenc, I would ask the authors to please include this information.

We now provide absolute population sizes and include the number of resuspended *M. xanthus* cells added to *P. fluorescens.* We apologize for not having included these details previously.

Figure S6: As requested, the authors have added an image showing the drop-collapse assay mentioned in the discussion. However, in the provided image and legend, I can't make out what the figure is supposed to show. From the mentioned reference, I understand that it should be liquid drops on a lid of a 96-well plate (?) I would please ask for a better quality image and a bit more information in the figure legend.

We experienced issues when converting our word document into pdf which resulted in modifications of our figures. We apologize and now provide the correct versions of figures S6, 4 and 5.
